# 4,6-Di-*tert*-butyl-2,8-dimeth­oxy­dibenzo[*b*,*d*]furan

**DOI:** 10.1107/S1600536811048379

**Published:** 2011-11-19

**Authors:** Dayeon Chung, Enkhzul Otgonbaatar, Seok Hwan Son, Minchul Chung, Chee-Hun Kwak

**Affiliations:** a Gwangju Science High School, Gwangju 500-480, Republic of Korea; b Department of Chemistry, Sunchon National University, Sunchon 540-742, Republic of Korea; c Department of Chemical Engineering, Sunchon National University, Sunchon 540-742, Republic of Korea

## Abstract

In the title compound, C_22_H_28_O_3_, the dihedral angle between the benzene rings is 3.47 (13)° and the five-membered furan ring is essentially planar with a largest deviation of 0.0052 (14) Å. The C*sp* ^2^—C*sp* ^2^ bond length between the two benzene rings [1.443 (3) Å] is considerably shorter than those between the benzene and tertiary C atoms [1.538 (3) and 1.530 (3) Å], which are *sp* ^2^–*sp* ^3^ hybridized. C—H⋯π inter­actions involving the furan and benzene rings are found in the crystal structure.

## Related literature

For the synthesis of the title compound, see: Hewgill & Hewitt (1967 ▶); Butsgan *et al.* (1989 ▶); Malkowsky *et al.* (2006 ▶). For a related structure, see: Du & Wang (2009 ▶).
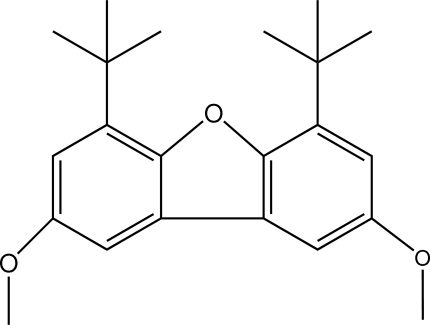


## Experimental

### 

#### Crystal data

C_22_H_28_O_3_
*M* *_r_* = 340.44
Monoclinic, 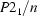

*a* = 15.631 (3) Å
*b* = 8.2487 (14) Å
*c* = 16.000 (3) Å
β = 105.438 (5)°
*V* = 1988.5 (6) Å^3^
*Z* = 4
Mo *K*α radiation
μ = 0.07 mm^−1^
*T* = 100 K
0.5 × 0.4 × 0.2 mm

#### Data collection

Rigaku R-AXIS RAPID II-S diffractometer
Absorption correction: multi-scan (*RAPID-AUTO*; Rigaku, 2008 ▶) *T*_min_ = 0.965, *T*_max_ = 0.985
18260 measured reflections
4563 independent reflections
2123 reflections with *I* > 2σ(*I*)
*R*_int_ = 0.107

#### Refinement

*R*[*F* ^2^ > 2σ(*F* ^2^)] = 0.076
*wR*(*F* ^2^) = 0.229
*S* = 0.99
4563 reflections
227 parameters
H-atom parameters not refined
Δρ_max_ = 0.24 e Å^−3^
Δρ_min_ = −0.21 e Å^−3^

### 

Data collection: *RAPID-AUTO* (Rigaku, 2008 ▶); cell refinement: *RAPID-AUTO*; data reduction: *RAPID-AUTO*; program(s) used to solve structure: *SHELXS97* (Sheldrick, 2008 ▶); program(s) used to refine structure: *SHELXL97* (Sheldrick, 2008 ▶); molecular graphics: *ORTEP-3* (Farrugia, 1997 ▶); software used to prepare material for publication: *WinGX* (Farrugia, 1999 ▶).

## Supplementary Material

Crystal structure: contains datablock(s) I, global. DOI: 10.1107/S1600536811048379/bq2314sup1.cif

Structure factors: contains datablock(s) I. DOI: 10.1107/S1600536811048379/bq2314Isup2.hkl

Supplementary material file. DOI: 10.1107/S1600536811048379/bq2314Isup3.cml

Additional supplementary materials:  crystallographic information; 3D view; checkCIF report

## Figures and Tables

**Table 1:** Hydrogen-bond geometry (Å, °) *Cg*1 and *Cg*2 are the centroids of the C5–C8/C11/C12 and O1/C9–C12 rings, respectively.

*D*—H⋯*A*	*D*—H	H⋯*A*	*D*⋯*A*	*D*—H⋯*A*
C15—H15*A*⋯*Cg*1^i^	0.96	2.98	3.580 (3)	121
C15—H15*B*⋯*Cg*2^i^	0.96	2.65	3.200 (3)	117
C22—H15*A*⋯*Cg*2^ii^	0.96	2.99	3.872 (4)	152

Symmetry codes: (i) 
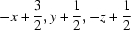
; (ii) 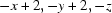.

**Fig. 1.**
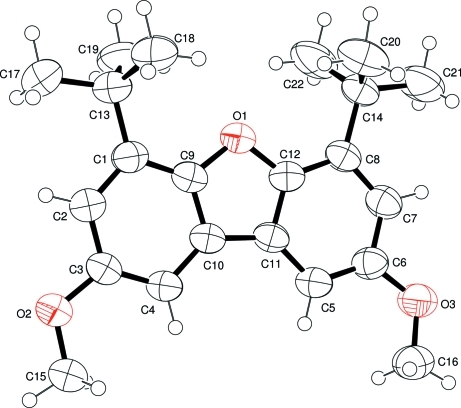
The structure of the title compound with displacement ellipsoids drawn at 50% probability level.

**Fig. 2.**
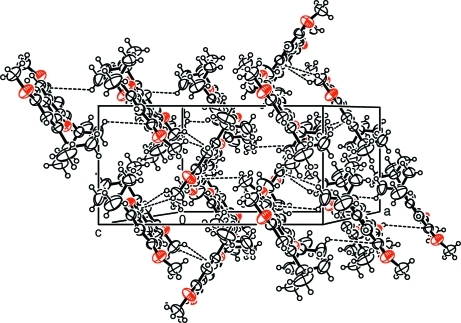
A packing diagram of the title compound. Dashed lines represent C—H···π interactions.

## supplementary crystallographic information

### Comment

Oxidative coupling of phenyl is a useful synthetic route for the synthesis of natural products (Malkowsky *et al.*, 2006). 2-*tert*-Butyl-4-methoxyphenol (BHA) is well kwon as an antioxidant and oxidative coupling of it produces di-BHA and benzofuran derivative by the various method of preparation (Hewgill & Hewitt, 1967; Butsgan *et al.*, 1989). The single-crystal structure of di-BHA was reported but that of benzofuran derived from BHA has not been investigated. Here we describe the structure of title compound obtained from pyrolysis method.

The title compound, C_22_H_29_O_3_, forms tricycle adjoined two benzene skeleton in C10 and C11, and C9 and C12 through O1 (Fig. 1). All atoms lies in almost a plane, the dihedral angle between two benzene skeletons is 3.47 (13)° and 5-membered furan ring is a plane with the largest deviation of 0.0052 (14) Å. The bond distance of C10—C11 [1.443 (3) Å] in *sp*^2^ –*sp*^2^ hybridization, which connects two benzene skeleton, is considerably shorter than that of C1—C13 [1.538 (3) Å] or C8—C14 [1.530 (3) Å] in *sp*^2^-*sp*^3^ hybridization. C15—H15A···π (3/2 - *x*, 1/2 + *y*, 1/2 - *z*) interaction involving the benzene ring (C5-C8/C11-C12) and, C15—H15B···π and C22—H22A···π (2 - *x*, 2 - *y*, -*z*) interactions involing furan ring (O1/C9—C12) are found in the crystal structure (Fig. 2 and Table 1). No classical hydrogen bond is found in the crystal structure.

### Experimental

A mixture of BHA (1.20 g, 6.65 mmol) and iron (0.95 g) and copper (0.89 g) powder in a Schlenk tube was heated under argon gas until BHA was melt and this mixture keep *ca* 170°C for 24 h. Dissolving the product with 30 ml CH_2_Cl_2_ for 3 times, the solution was chromatographed on Al_2_O_3_ eluting with CH_2_Cl_2_/n-hexane(1:1) to afford the title compound. Single crystals of the compound for X-ray analysis were obtained by recrystallization from CH_2_Cl_2_/n-hexane (1:1) at -20°C. ^13^C-NMR (THF-*d*^5^) δ 29.245 (C(*C*H_3_)_3_), 32.843 ((O-*C*H_3_), 54.707 (*C*(CH_3_)_3_), 11.825, 113.253, 128.572, 139.625, 145.875, 153.619 (Phenyl). ESI-MS (*M*/*z*) C_22_H_28_O_3_; Observed (cal'd): [*M*+H]^+^ = 341.2186 (340.46).

### Refinement

The H atoms were positioned geometrically and ride on their respective parent atoms. C—H Distance is 0.93 Å (CH, *sp*^2^) with *U*_iso_ = 1.2*U*_eq_(C) and 0.96 Å (CH_3_) with *U*_iso_ = 1.5*U*_eq_(C).

### Crystal data

**Table:**

C_22_H_28_O_3_	*F*(000) = 736
*M**_r_* = 340.44	*D*_x_ = 1.137 Mg m^−^^3^
Monoclinic, *P*2_1_/*n*	Mo *K*α radiation, λ = 0.71073 Å
*a* = 15.631 (3) Å	Cell parameters from 18260 reflections
*b* = 8.2487 (14) Å	θ = 3.2–27.5°
*c* = 16.000 (3) Å	µ = 0.07 mm^−^^1^
β = 105.438 (5)°	*T* = 100 K
*V* = 1988.5 (6) Å^3^	Block, brown
*Z* = 4	0.5 × 0.4 × 0.2 mm

### Data collection

**Table:**

Rigaku R-AXIS RAPID II-S diffractometer	4563 independent reflections
Radiation source: fine-focus sealed tube	2123 reflections with *I* > 2σ(*I*)
graphite	*R*_int_ = 0.107
ω scans	θ_max_ = 27.5°, θ_min_ = 3.2°
Absorption correction: multi-scan (*RAPID-AUTO*; Rigaku, 2008)	*h* = −20→20
*T*_min_ = 0.965, *T*_max_ = 0.985	*k* = −9→10
18260 measured reflections	*l* = −20→20

### Refinement

**Table:**

Refinement on *F*^2^	Primary atom site location: structure-invariant direct methods
Least-squares matrix: full	Secondary atom site location: difference Fourier map
*R*[*F*^2^ > 2σ(*F*^2^)] = 0.076	Hydrogen site location: inferred from neighbouring sites
*wR*(*F*^2^) = 0.229	H-atom parameters not refined
*S* = 0.99	*w* = 1/[σ^2^(*F*_o_^2^) + (0.1073*P*)^2^] where *P* = (*F*_o_^2^ + 2*F*_c_^2^)/3
4563 reflections	(Δ/σ)_max_ = 0.009
227 parameters	Δρ_max_ = 0.24 e Å^−^^3^
0 restraints	Δρ_min_ = −0.21 e Å^−^^3^

### Special details

**Table:**

Geometry. All e.s.d.'s (except the e.s.d. in the dihedral angle between two l.s. planes) are estimated using the full covariance matrix. The cell e.s.d.'s are taken into account individually in the estimation of e.s.d.'s in distances, angles and torsion angles; correlations between e.s.d.'s in cell parameters are only used when they are defined by crystal symmetry. An approximate (isotropic) treatment of cell e.s.d.'s is used for estimating e.s.d.'s involving l.s. planes.
Refinement. Refinement of *F*^2^ against ALL reflections. The weighted *R*-factor *wR* and goodness of fit *S* are based on *F*^2^, conventional *R*-factors *R* are based on *F*, with *F* set to zero for negative *F*^2^. The threshold expression of *F*^2^ > σ(*F*^2^) is used only for calculating *R*-factors(gt) *etc*. and is not relevant to the choice of reflections for refinement. *R*-factors based on *F*^2^ are statistically about twice as large as those based on *F*, and *R*- factors based on ALL data will be even larger.

### Fractional atomic coordinates and isotropic or equivalent isotropic displacement parameters (Å^2^)

**Table:**

	*x*	*y*	*z*	*U*_iso_*/*U*_eq_	
O1	0.90422 (11)	0.83006 (19)	0.11113 (11)	0.0570 (5)	
O2	0.87127 (14)	1.1093 (2)	0.41186 (12)	0.0787 (6)	
C11	0.81556 (16)	1.0573 (3)	0.08804 (16)	0.0515 (6)	
C10	0.84681 (16)	1.0295 (3)	0.18023 (17)	0.0524 (6)	
C12	0.85219 (16)	0.9345 (3)	0.04927 (17)	0.0528 (6)	
O3	0.68536 (15)	1.2535 (2)	−0.11118 (13)	0.0805 (6)	
C5	0.75816 (17)	1.1710 (3)	0.03733 (17)	0.0585 (7)	
H5	0.7323	1.2532	0.0621	0.070*	
C4	0.83376 (17)	1.1096 (3)	0.25353 (17)	0.0560 (6)	
H4	0.7990	1.2024	0.2483	0.067*	
C3	0.87496 (17)	1.0440 (3)	0.33359 (17)	0.0592 (7)	
C1	0.93919 (17)	0.8188 (3)	0.27084 (18)	0.0589 (7)	
C7	0.78180 (18)	1.0324 (3)	−0.08857 (18)	0.0633 (7)	
H7	0.7697	1.0287	−0.1487	0.076*	
C9	0.89907 (17)	0.8908 (3)	0.19084 (17)	0.0551 (6)	
C6	0.74191 (18)	1.1549 (3)	−0.05084 (18)	0.0610 (7)	
C8	0.83866 (17)	0.9163 (3)	−0.03992 (17)	0.0563 (6)	
C14	0.87778 (18)	0.7790 (3)	−0.08242 (18)	0.0617 (7)	
C2	0.92504 (17)	0.9010 (3)	0.34121 (18)	0.0633 (7)	
H2	0.9499	0.8595	0.3964	0.076*	
C13	0.9922 (2)	0.6597 (3)	0.2794 (2)	0.0734 (8)	
C15	0.8178 (2)	1.2486 (4)	0.4088 (2)	0.0791 (9)	
H15A	0.8197	1.2819	0.4668	0.119*	
H15B	0.7577	1.2238	0.3779	0.119*	
H15C	0.8397	1.3345	0.3797	0.119*	
C18	0.9331 (2)	0.5255 (3)	0.2286 (2)	0.0874 (10)	
H18A	0.9123	0.5566	0.1688	0.131*	
H18B	0.8834	0.5088	0.2521	0.131*	
H18C	0.9667	0.4269	0.2330	0.131*	
C22	0.9777 (2)	0.7683 (5)	−0.0432 (3)	0.1029 (13)	
H22A	1.0051	0.8670	−0.0547	0.154*	
H22B	1.0010	0.6787	−0.0686	0.154*	
H22C	0.9900	0.7524	0.0183	0.154*	
C16	0.6289 (2)	1.3589 (4)	−0.0816 (2)	0.0826 (9)	
H16A	0.5937	1.4200	−0.1295	0.124*	
H16B	0.6639	1.4317	−0.0392	0.124*	
H16C	0.5907	1.2968	−0.0558	0.124*	
C19	1.0732 (2)	0.6880 (4)	0.2426 (3)	0.1032 (12)	
H19A	1.0531	0.7258	0.1838	0.155*	
H19B	1.1050	0.5881	0.2439	0.155*	
H19C	1.1116	0.7676	0.2771	0.155*	
C20	0.8357 (2)	0.6196 (4)	−0.0658 (2)	0.0952 (11)	
H20A	0.8430	0.6067	−0.0047	0.143*	
H20B	0.8640	0.5312	−0.0870	0.143*	
H20C	0.7736	0.6207	−0.0954	0.143*	
C21	0.8591 (3)	0.8028 (5)	−0.1807 (2)	0.1160 (14)	
H21A	0.8840	0.9041	−0.1923	0.174*	
H21B	0.7962	0.8035	−0.2063	0.174*	
H21C	0.8855	0.7157	−0.2049	0.174*	
C17	1.0251 (3)	0.6062 (4)	0.3745 (2)	0.1147 (14)	
H17A	1.0570	0.5060	0.3778	0.172*	
H17B	0.9752	0.5913	0.3981	0.172*	
H17C	1.0636	0.6880	0.4071	0.172*	

### Atomic displacement parameters (Å^2^)

**Table:**

	*U*^11^	*U*^22^	*U*^33^	*U*^12^	*U*^13^	*U*^23^
O1	0.0590 (10)	0.0531 (10)	0.0617 (12)	0.0046 (8)	0.0209 (9)	−0.0053 (8)
O2	0.0938 (15)	0.0840 (13)	0.0594 (12)	0.0314 (11)	0.0220 (11)	−0.0055 (10)
C11	0.0529 (13)	0.0463 (12)	0.0617 (16)	−0.0036 (10)	0.0262 (12)	−0.0047 (11)
C10	0.0525 (14)	0.0471 (12)	0.0619 (16)	−0.0012 (10)	0.0225 (12)	−0.0034 (11)
C12	0.0525 (13)	0.0456 (12)	0.0644 (16)	−0.0011 (10)	0.0230 (12)	0.0009 (12)
O3	0.0993 (15)	0.0751 (13)	0.0708 (13)	0.0233 (12)	0.0293 (12)	0.0033 (10)
C5	0.0650 (16)	0.0509 (13)	0.0654 (17)	0.0050 (12)	0.0275 (13)	−0.0009 (12)
C4	0.0599 (15)	0.0498 (13)	0.0628 (16)	0.0045 (11)	0.0240 (13)	−0.0023 (12)
C3	0.0636 (16)	0.0601 (15)	0.0578 (16)	0.0047 (12)	0.0226 (13)	−0.0073 (13)
C1	0.0559 (14)	0.0522 (14)	0.0683 (18)	0.0053 (11)	0.0159 (13)	−0.0020 (13)
C7	0.0722 (17)	0.0631 (16)	0.0608 (16)	−0.0004 (14)	0.0286 (14)	−0.0035 (13)
C9	0.0559 (14)	0.0493 (13)	0.0631 (16)	−0.0016 (11)	0.0209 (12)	−0.0076 (12)
C6	0.0662 (16)	0.0562 (14)	0.0654 (17)	0.0056 (12)	0.0258 (14)	0.0056 (13)
C8	0.0588 (15)	0.0542 (14)	0.0615 (16)	−0.0036 (12)	0.0261 (13)	−0.0076 (12)
C14	0.0662 (16)	0.0605 (15)	0.0666 (18)	−0.0005 (13)	0.0317 (14)	−0.0107 (13)
C2	0.0664 (16)	0.0594 (15)	0.0632 (16)	0.0100 (13)	0.0158 (13)	−0.0003 (13)
C13	0.079 (2)	0.0594 (16)	0.078 (2)	0.0187 (14)	0.0146 (16)	−0.0048 (15)
C15	0.085 (2)	0.084 (2)	0.0688 (19)	0.0247 (16)	0.0222 (16)	−0.0115 (16)
C18	0.105 (2)	0.0525 (16)	0.101 (3)	0.0133 (16)	0.022 (2)	0.0001 (16)
C22	0.069 (2)	0.124 (3)	0.125 (3)	0.0018 (19)	0.041 (2)	−0.056 (2)
C16	0.092 (2)	0.0692 (18)	0.087 (2)	0.0197 (16)	0.0261 (18)	0.0021 (17)
C19	0.069 (2)	0.095 (2)	0.143 (4)	0.0249 (18)	0.024 (2)	−0.020 (2)
C20	0.110 (3)	0.0655 (19)	0.121 (3)	−0.0122 (18)	0.051 (2)	−0.0273 (19)
C21	0.176 (4)	0.109 (3)	0.078 (2)	0.046 (3)	0.059 (3)	−0.009 (2)
C17	0.151 (4)	0.086 (2)	0.086 (2)	0.054 (2)	−0.004 (2)	0.002 (2)

### Geometric parameters (Å, °)

**Table:**

O1—C9	1.393 (3)	C13—C18	1.529 (4)
O1—C12	1.398 (3)	C13—C17	1.536 (4)
O2—C3	1.378 (3)	C13—C19	1.549 (5)
O2—C15	1.413 (3)	C15—H15A	0.9600
C11—C12	1.389 (3)	C15—H15B	0.9600
C11—C5	1.398 (3)	C15—H15C	0.9600
C11—C10	1.443 (3)	C18—H18A	0.9600
C10—C9	1.390 (3)	C18—H18B	0.9600
C10—C4	1.407 (3)	C18—H18C	0.9600
C12—C8	1.394 (3)	C22—H22A	0.9600
O3—C6	1.386 (3)	C22—H22B	0.9600
O3—C16	1.408 (3)	C22—H22C	0.9600
C5—C6	1.372 (4)	C16—H16A	0.9600
C5—H5	0.9300	C16—H16B	0.9600
C4—C3	1.382 (4)	C16—H16C	0.9600
C4—H4	0.9300	C19—H19A	0.9600
C3—C2	1.403 (3)	C19—H19B	0.9600
C1—C2	1.382 (4)	C19—H19C	0.9600
C1—C9	1.399 (4)	C20—H20A	0.9600
C1—C13	1.538 (3)	C20—H20B	0.9600
C7—C8	1.395 (4)	C20—H20C	0.9600
C7—C6	1.405 (4)	C21—H21A	0.9600
C7—H7	0.9300	C21—H21B	0.9600
C8—C14	1.530 (3)	C21—H21C	0.9600
C14—C22	1.523 (4)	C17—H17A	0.9600
C14—C20	1.524 (4)	C17—H17B	0.9600
C14—C21	1.533 (4)	C17—H17C	0.9600
C2—H2	0.9300		
			
C9—O1—C12	105.20 (18)	C1—C13—C19	108.2 (2)
C3—O2—C15	117.0 (2)	O2—C15—H15A	109.5
C12—C11—C5	120.5 (2)	O2—C15—H15B	109.5
C12—C11—C10	105.8 (2)	H15A—C15—H15B	109.5
C5—C11—C10	133.6 (2)	O2—C15—H15C	109.5
C9—C10—C4	119.7 (2)	H15A—C15—H15C	109.5
C9—C10—C11	106.5 (2)	H15B—C15—H15C	109.5
C4—C10—C11	133.8 (2)	C13—C18—H18A	109.5
C11—C12—C8	124.6 (2)	C13—C18—H18B	109.5
C11—C12—O1	111.4 (2)	H18A—C18—H18B	109.5
C8—C12—O1	124.0 (2)	C13—C18—H18C	109.5
C6—O3—C16	117.9 (2)	H18A—C18—H18C	109.5
C6—C5—C11	116.5 (2)	H18B—C18—H18C	109.5
C6—C5—H5	121.7	C14—C22—H22A	109.5
C11—C5—H5	121.7	C14—C22—H22B	109.5
C3—C4—C10	117.0 (2)	H22A—C22—H22B	109.5
C3—C4—H4	121.5	C14—C22—H22C	109.5
C10—C4—H4	121.5	H22A—C22—H22C	109.5
O2—C3—C4	124.6 (2)	H22B—C22—H22C	109.5
O2—C3—C2	114.0 (2)	O3—C16—H16A	109.5
C4—C3—C2	121.4 (2)	O3—C16—H16B	109.5
C2—C1—C9	114.0 (2)	H16A—C16—H16B	109.5
C2—C1—C13	123.1 (2)	O3—C16—H16C	109.5
C9—C1—C13	122.9 (2)	H16A—C16—H16C	109.5
C8—C7—C6	123.0 (3)	H16B—C16—H16C	109.5
C8—C7—H7	118.5	C13—C19—H19A	109.5
C6—C7—H7	118.5	C13—C19—H19B	109.5
C10—C9—O1	111.1 (2)	H19A—C19—H19B	109.5
C10—C9—C1	124.6 (2)	C13—C19—H19C	109.5
O1—C9—C1	124.3 (2)	H19A—C19—H19C	109.5
C5—C6—O3	124.7 (2)	H19B—C19—H19C	109.5
C5—C6—C7	122.0 (3)	C14—C20—H20A	109.5
O3—C6—C7	113.3 (2)	C14—C20—H20B	109.5
C12—C8—C7	113.4 (2)	H20A—C20—H20B	109.5
C12—C8—C14	124.4 (2)	C14—C20—H20C	109.5
C7—C8—C14	122.1 (2)	H20A—C20—H20C	109.5
C22—C14—C20	108.7 (3)	H20B—C20—H20C	109.5
C22—C14—C8	110.3 (2)	C14—C21—H21A	109.5
C20—C14—C8	108.6 (2)	C14—C21—H21B	109.5
C22—C14—C21	108.9 (3)	H21A—C21—H21B	109.5
C20—C14—C21	108.4 (3)	C14—C21—H21C	109.5
C8—C14—C21	111.8 (2)	H21A—C21—H21C	109.5
C1—C2—C3	123.3 (3)	H21B—C21—H21C	109.5
C1—C2—H2	118.4	C13—C17—H17A	109.5
C3—C2—H2	118.4	C13—C17—H17B	109.5
C18—C13—C17	108.3 (3)	H17A—C17—H17B	109.5
C18—C13—C1	109.6 (2)	C13—C17—H17C	109.5
C17—C13—C1	111.3 (2)	H17A—C17—H17C	109.5
C18—C13—C19	110.2 (3)	H17B—C17—H17C	109.5
C17—C13—C19	109.2 (3)		

### Hydrogen-bond geometry (Å, °)

**Table:**

*Cg*1 and *Cg*2 are the centroids of the C5–C8/C11/C12 and O1/C9–C12 rings, respectively.

**Table:**

*D*—H···*A*	*D*—H	H···*A*	*D*···*A*	*D*—H···*A*
C15—H15*A*···*Cg*1^i^	0.96	2.98	3.580 (3)	121
C15—H15*B*···*Cg*2^i^	0.96	2.65	3.200 (3)	117
C22—H15*A*···*Cg*2^ii^^ii^	0.96	2.99	3.872 (4)	152

Symmetry codes: (i) −*x*+3/2, *y*+1/2, −*z*+1/2; (ii) −*x*+2, −*y*+2, −*z*.
